# The genetic contribution of the X chromosome in age-related hearing loss

**DOI:** 10.3389/fgene.2023.1106328

**Published:** 2023-02-21

**Authors:** Elnaz Naderi, Diana M. Cornejo-Sanchez, Guangyou Li, Isabelle Schrauwen, Gao T. Wang, Andrew T. Dewan, Suzanne M. Leal

**Affiliations:** ^1^ Center for Statistical Genetics, Gertrude H. Sergievsky Center, and the Department of Neurology, Columbia University Medical Center, New York, NY, United States; ^2^ Department of Chronic Disease Epidemiology and Center for Perinatal, Pediatric and Environmental Epidemiology, Yale School of Public Health, New Haven, CT, United States; ^3^ Taub Institute for Alzheimer’s Disease and the Aging Brain, Columbia University Medical Center, New York, NY, United States

**Keywords:** age-related hearing loss, *MAP7D2*, *LOC101928437*, *ZNF185*, UK Biobank, X chromosome

## Abstract

Age-related (AR) hearing loss (HL) is the most common sensory impairment with heritability of 55%. The aim of this study was to identify genetic variants on chromosome X associated with ARHL through the analysis of data obtained from the UK Biobank. We performed association analysis between self-reported measures of HL and genotyped and imputed variants on chromosome X from ∼460,000 white Europeans. We identified three loci associated with ARHL with a genome-wide significance level (p < 5 × 10^−8^), *ZNF185* (rs186256023, *p* = 4.9 × 10^−10^) and *MAP7D2* (rs4370706, *p* = 2.3 × 10^−8^) in combined analysis of males and females, and *LOC101928437* (rs138497700, *p* = 8.9 × 10^−9^) in the sex-stratified analysis of males. *In-silico* mRNA expression analysis showed *MAP7D2* and *ZNF185* are expressed in mice and adult human inner ear tissues, particularly in the inner hair cells. We estimated that only a small amount of variation of ARHL, 0.4%, is explained by variants on the X chromosome. This study suggests that although there are likely a few genes contributing to ARHL on the X chromosome, the role that the X chromosome plays in the etiology of ARHL may be limited.

## 1 Introduction

Age-related (AR) hearing Loss (HL), as known as presbycusis, is the most frequent sensory impairment affecting 25%–40% of individuals ≥65 years of age (Wolber et al., 2012). The prevalence of ARHL increases with age (Salvi et al., 2018), with males having a higher prevalence than females (Van Eyken et al., 2007). With lifespan increasing, especially in developed countries, the impact of ARHL will continue to increase.

ARHL is a complex disorder impacted by risk factors such as sex, noise exposure, ototoxic medications, and genetic susceptibility (Wiley et al., 2008; Bowl & Dawson, 2019). ARHL is also associated with several comorbidities, including cognitive decline, depression, diabetes, and hypertension (Yuan et al., 2018; Ponticorvo et al., 2019). Twin and family studies have shown that the heritability of ARHL ranges from 0.35 to 0.55 (Christensen et al., 2001; Duan et al., 2019). Genome-wide association studies (GWAS) have identified several genetic loci associated with ARHL [e.g., *ARHGEF28* (MIM: 612790), *CHMP4C* (MIM: 610899), *ZNF318* (MIM: 617512)] (Hoffmann et al., 2016; Wells et al., 2019).

To date, six early-onset non-syndromic HL genes and 21 syndromic HL genes have been identified on the X chromosome (Corvino et al., 2018); however, no X-linked genes or loci have been reported to be involved in etiology of ARHL. Although the X chromosome is the eighth largest chromosome and contains ∼5% of human genes (König et al., 2014) it is usually excluded from GWAS analyses (Wise et al., 2013).

Here we focused on examining the role of variants on chromosome X in ARHL through the analysis of genotype array and imputed data and self-reported measures of HL obtained from ∼460,000 white-European UK Biobank volunteers.

In this first report of the role of the X chromosome in ARHL, associations were identified between ARHL and variants near *ZNF185, MAP7D2*, and *LOC101928437*. *In-silico* analysis revealed inner ear expression of *MAP7D2/Map7d2* and *ZNF185/Zfp185* in mice and humans, particularly for the inner ear hair cells. Additionally, the estimated heritability of ARHL due to single nucleotide variants (SNVs) on the X chromosome with a minor allele frequency (MAF) > 0.01 is 0.4%.

## 2 Materials and methods

### 2.1 Data access and ethical approval

This research was conducted using the UK Biobank Resource (application numbers 32285 and 36,827). The UK Biobank study was conducted under generic approval from the National Health Services’ National Research Ethics Service. The present analyses were approved by the Institutional Review Boards at Yale University (2000026836) and Columbia University (AAAS3494).

### 2.2 Study population

The UK Biobank is a cohort of ∼500,000 volunteers between 40 and 69 years-of-age at the time of recruitment from 2006 to 2010 and are being followed-up for at least 20 years (Sudlow et al., 2015). Detailed information about the UK Biobank can be found elsewhere (Sudlow et al., 2015; Bycroft et al., 2018).

### 2.3 Genotyping, imputation, and quality control

The UK Biobank samples were genotyped on two arrays: Affymetrix UK BiLEVE Axiom array (50,000 individuals) and the Affymetrix UK Biobank Axiom array (450,000 individuals) (Bycroft et al., 2018). The two arrays share 733,322 autosomal and 20,214 X chromosome variants. Genotypes were phased using SHAPEIT3 (O’Connell et al., 2016) and imputation was performed with IMPUTE4 (Bycroft et al., 2018) using reference data from the Haplotype Reference Consortium and UK10K (Huang et al., 2015; McCarthy et al., 2016). The pseudoautosomal regions (PARs) and non-pseudoautosomal region (non-PAR) were phased and imputed independently. Genotyping, haplotype phasing, and imputation have been previously described (Bycroft et al., 2018).

Individuals with putative sex chromosome aneuploidy, inconsistent sex (reported sex did not match genetic sex) or were missing >3% of their genotype array data were removed. Analysis was restricted to individuals of white-European ancestry (N = 459,267) based upon principal components analysis. The genotype variants with call rate >95%, Hardy Weinberg Equilibrium *p*-value < 5.0 × 10^−8^ (estimated in non-PAR region in females and the variants out of HWE were removed in both females and males), and minor allele frequency (MAF) > 0.001 were used for whole-genome ridge regression (*N* = 442,313, Supplementary Table S1) implemented in REGENIE (Mbatchou et al., 2021). Principal components (PCs) of genetic data to include in association analysis were generated using a subset of genotyped markers (*N* = 144,905) that were pruned for linkage disequilibrium (LD) (*r* ^2^ > 0.1) (Supplementary Table S1).

SNVs on the X chromosome obtain from the genotype arrays that did not deviate from HWE and had a MAF>0.001 (1,207 PAR and 11,653 non-PAR) were analyzed. Imputed X chromosome SNVs and insertion/deletions (InDels) with a MAF>0.001 and INFO Score ≥0.3 (45,519 PAR and 2,050,039 non-PAR) were also analyzed. To estimate the contribution of common variants on X chromosome to heritability of ARHL we analyzed variants obtained from the genotype array data with MAF>0.01 in the non-PAR region (*N* = 18,773).

### 2.4 Phenotype definition

Using ICD10/ICD9 codes and self-reports, individuals with chronic otitis media, salpingitis, mastoiditis, otosclerosis, Meniere’s disease, deafness, labyrinthitis, conductive HL, ototoxic HL, head, ear or neck trauma, stroke, encephalitis, meningitis, or facial nerve disorders were excluded from the analysis. Additionally, individuals with unilateral/bilateral sensorineural or mixed conductive HL were excluded if they were diagnosed <55 years-of-age or did not have an age-of-diagnosis (Supplementary Table S2).

Four different case status were determined based on the response to a touchscreen questionnaire: 1) ***H-aid*** self-reported hearing aid use (f.3393: “Do you use a hearing aid most of the time?“); 2) ***H-diff*** self-reported hearing difficulty (f.2247: “Do you have any difficulty with your hearing?”); 3) ***H-noise*** self-reported hearing difficulty with background noise (f.2257: “Do you find it difficult to follow a conversation if there is background noise, e.g., TV, radio, children playing)?”; and 4) ***H-both*** individuals with both *H-diff* and *H-noise*. Individuals who provided inconsistent answers for *H-aid*, H-diff, or *H-noise* (e.g., reported *H-diff* on the first visit but not the second visit), or did not provide definite answers, (e.g., answered “Do not know”) were excluded (Supplementary Table S1). In the analysis age is defined for cases, when they answered “Yes” to a specific ARHL question for the first time during the assessments. We used a common set of controls without any hearing-related phenotypes. For the controls, age at last assessment was used in the analysis.

Information on the number of individuals available for analysis for each phenotype (*H-aid*, *H-diff*, *H-noise*, and *H-both*) as well as information on the age distribution among males and females can be found in Supplementary Table S3.

### 2.5 Association analysis

Univariate analysis was performed using a generalized linear mixed model (GLMM) as implemented in REGENIE 2.2.4 (Mbatchou et al., 2021). REGENIE uses a multi-stage approach by initially fitting a whole-genome regression model *via* ridge regression to estimate the polygenic background accounting for population structure and relatedness. A firth correction for binary traits is applied to calibrate the model in the presence of low frequency variants and unbalanced case-control ratios, as is the case for this analysis (i.e., *H-aid* 1:15 cases to controls). In step 2, score tests for association were performed for each genotyped and imputed variants with MAF >0.001 on the X chromosome. Genotypes for PAR X chromosomal variants were coded {0, 1, 2} for both males and females, whereas for non-PAR X chromosomal variants males were coded as {0, 2}. Imputed dosages were used for imputed genotypes. An additive effect model was used in the association analysis to adjust for age, sex, array (BiLEVE and Axiom), and two PCs. For the non-PAR variants, a sex-stratified analysis was also performed using the same model.

### 2.6 Heritability

Heritability was estimated for variants on the X chromosome using GCTA implementing a restricted maximum likelihood (REML) approach (Yang et al., 2011). The genetic relatedness matrix (GRM) was calculated using X chromosome genotyped variants. Age, sex, array type (BiLEVE and Axiom), and two PCs were included as covariates.

### 2.7 *In-silico* mRNA expression analysis

Human and mouse inner ear gene expressions were assessed *in silico*. To study expression during mouse inner ear development, an otic progenitor cells dataset was obtained from the Shared Harvard Inner-Ear Laboratory Database (SHIELD) (Shen et al., 2015). The Expression Analysis Resource (gEAR) was also used to visualize single cell RNA-seq data of the cochlear epithelium during four mouse developmental stages [embryonic day (E)14, E16, postnatal (P)1, and P7] (Kolla et al., 2020; Orvis et al., 2021) and of mouse otic neuronal lineages at three embryonic ages, embryonic day 9.5 (E9.5), E11.5, and E13.5 (Sun et al., 2022). Processing and normalization of expression values were performed using Seurat (Stuart et al., 2019) and single cells were grouped into cell clusters (Kolla et al., 2020; Sun et al., 2022). Finally, human inner ear expression data were obtained from RNA sequencing of inner ear tissue samples from three donor patients ages 45–60 (Schrauwen et al., 2016) and were processed and normalized using DESeq2 (Love et al., 2014).

## 3 Results

### 3.1 Case-control samples for association analysis

The number of cases and controls for each of the four ARHL traits are displayed in Supplementary Table S3. There is a higher proportion of male cases than female cases for each trait (two-sample z-test for proportions *p* < 2.23 × 10^−308^, Supplementary Table S3). Also, the mean age of males with HL was higher than females (two-sample independent *t*-test *p* < 2.81 × 10^−33^, Supplementary Table S3).

### 3.2 Detection of ARHL-associated variants

#### 3.2 1 Non-PAR region

**FIGURE 1:**
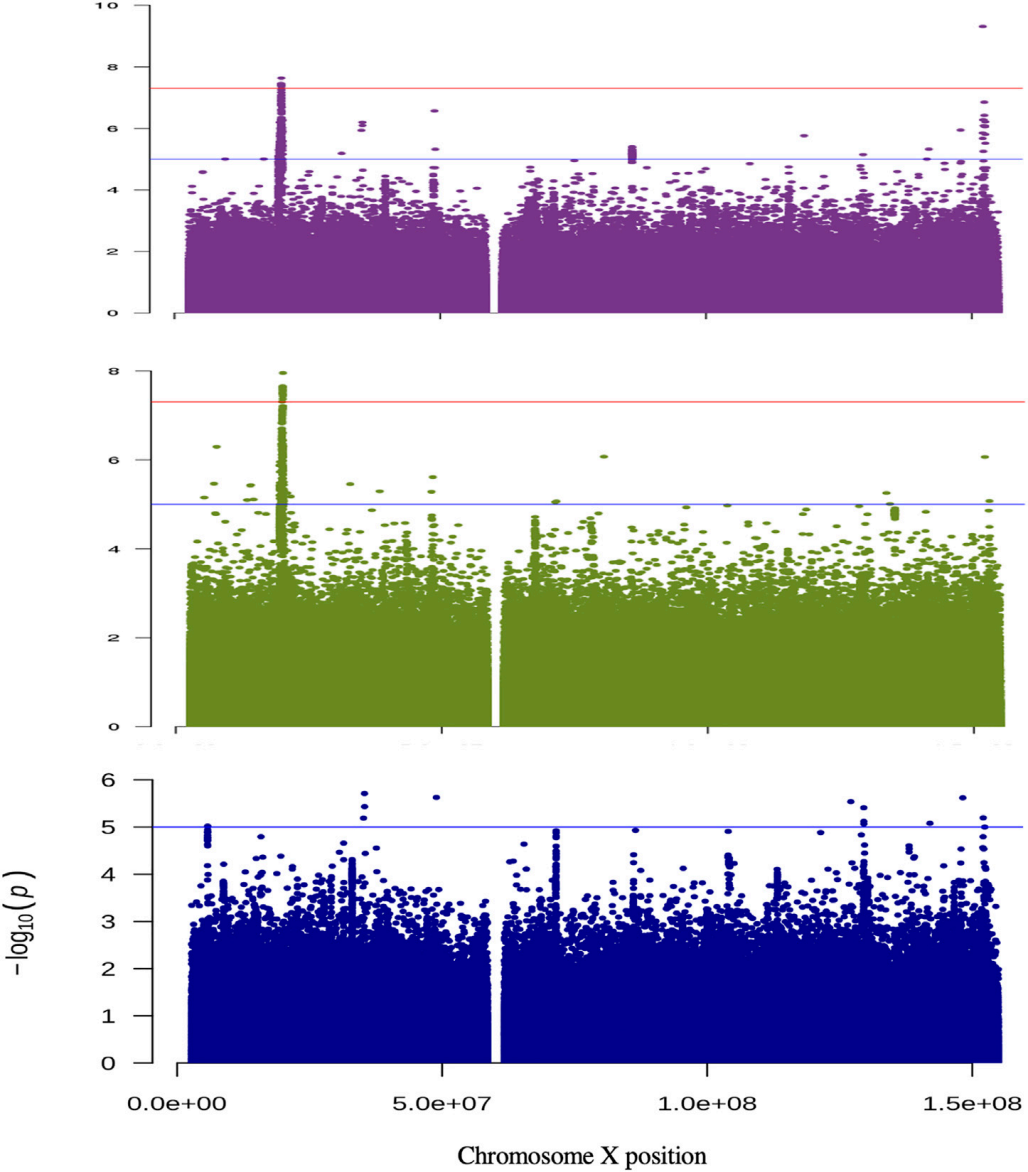
Manhattan plots for the analysis of non-pseudo autosomal region of the X chromosome. The results are shown for the analysis of *H-Both*: males and females (purple); females only (green) and males only (blue). The *y*-axis displays −log_10_ for each variant tested and the *X*-axis their position. The horizontal red and blue lines show the threshold for genome-wide significant (*p* = 5 × 10^−8^) and suggestive (*p* = 1 × 10^−5^) associations, respectively.

In the combined sex analysis for *H-both*, nine variants in two independent genomic regions reached genome-wide significance (Table 1 and Figure 1). The first region’s most significant variant is rs186256023 near *ZNF185* [*p* = 4.9 × 10^−10^, odds ratio (OR) = 1.08 with confidence interval (CI) = 1.062–1.105, MAF = 0.03] and the other genomic region’s most significant variant is rs4370706 near *MAP7D2* [*p* = 2.3 × 10^−8^, OR = 1.03 (CI = 1.010–1.051), MAF = 0.18]. For the other three traits, no genome-wide statistically significant variants were identified. Loci that displayed suggestive associations (5.0 × 10^−8^>*p*< 1.0 × 10^−5^) are shown in Supplementary Figure S1 and in Supplementary Tables S4–S7.

**TABLE 1:** Variants associated with age related hearing loss.

						Combined					Females				Males		
H-both	Variant	Pos	Locus	A1	Info	Freq	Beta (OR)	SE	P	N		Beta (OR)	P	N	Beta (OR)	P	N
	rs186256023	152067381	ZNF185	C	0.92	0.03	0.08 (1.083)	0.01	4.9E-10	330,558		0.11 (1.116)	8.6E-07	182,676	0.07 (1.072)	6.4E-06	147,882
	rs4370706	20,102,906	MAP7D2	T	0.99	0.18	0.03 (1.030)	0.01	2.3E-08	330,558		0.05 (1.051)	6.4E-08	182,676	0.02 (1.020)	2.5E-03	147,882
	rs7882991	20,054,650	MAP7D2	A	0.98	0.18	0.03 (1.030)	0.01	3.4E-08	330,558		0.05 (1.051)	3.3E-08	182,676	0.02 (1.020)	4.2E-03	147,882
	rs10081918	20,053,979	MAP7D2	T	0.98	0.18	0.03 (1.030)	0.01	3.6E-08	330,558		0.05 (1.051)	3.1E-08	182,676	0.02 (1.020)	4.4E-03	147,882
	rs5955880	20,084,743	MAP7D2	A	0.99	0.18	0.03 (1.030)	0.01	3.9E-08	330,558		0.06 (1.062)	2.2E-08	182,676	0.02 (1.020)	5.2E-03	147,882
	rs6527961	20,109,072	MAP7D2	A	0.99	0.18	0.03 (1.030)	0.01	3.9E-08	330,558		0.06 (1.062)	2.3E-08	182,676	0.02 (1.020)	5.1E-03	147,882
	rs12009531	20,074,441	MAP7D2	A	0.99	0.18	0.03 (1.030)	0.01	4.0E-08	330,558		0.06 (1.062)	2.4E-08	182,676	0.02 (1.020)	5.2E-03	147,882
	rs7062127	20,061,058	MAP7D2	G	0.98	0.18	0.03 (1.030)	0.01	4.0E-08	330,558		0.05 (1.051)	8.9E-08	182,676	0.02 (1.020)	3.6E-03	147,882
	rs60849679	20,116,215	MAP7D2	A	1	0.18	0.03 (1.030)	0.01	4.5E-08	330,558		0.05 (1.051)	3.7E-08	182,676	0.02 (1.020)	4.1E-03	147,882
	rs5955882	20,110,340	MAP7D2	T	0.98	0.18	0.03 (1.030)	0.01	5.7E-08	330,558		0.06 (1.062)	2.7E-08	182,676	0.02 (1.020)	6.4E-03	147,882
	rs7064198	20,101,842	MAP7D2	T	0.99	0.18	0.03 (1.030)	0.01	5.3E-08	330,558		0.06 (1.062)	2.9E-08	182,676	0.02 (1.020)	6.1E-03	147,882
H-aid	rs138497700	112,591,946	LOC101928437	G	0.83	0.003	2.00 (7.39)	0.58	4.1E-04	252,895		−1.17 (0.310)	0.228	148,632	2.6 (13.46)	8.9E-09	104,263
	rs141853173	112,449,530	LOC101928437	T	0.84	0.003	1.85 (6.36)	0.61	4.3E-04	252,895		−0.89 (0.411)	0.312	148,632	2.6 (13.46)	2.3E-08	104,263

Abbreviations; Pos, Position in x chromosome; A1, Effect allele; Info, Imputation quality; Freq, Frequency of effect allele based on combined samples; OR, Odd ratio; SE, Standard error; P, *p*-Value; N, Number of included individuals.

For the sex-stratified analysis of *H-both*, in females there are eight genome-wide significant variants within the *MAP7D2* genomic region, but none the variants had a *p* < 10^−4^ in males (Table 1 and Figure 1). Of the eight variants, rs5955880 that is near *MAP7D2* is the most significant [*p* = 2.2 × 10^−8^, OR = 1.06 (CI = 1.041–1.083)]. This same variant has a *p* = 5.2 × 10^−3^ in males [OR = 1.02 (CI = 1.006–1.034)].

For the sex-stratified analysis of *H-aid* there are two genome-wide significant variants in one genomic region in males, but none reached nominal significance in females (Table 1). The most significant SNV, rs138497700, near *LOC101928437* has a *p* = 8.9 × 10^−9^ [OR = 13.46 (CI = 5.92–30.61), MAF = 0.003]. This SNV has an OR = 0.31 (CI = 0.05–2.08) and *p* = 0.23 in females.

We found no genome-wide significant variants in the sex-stratified analyses for *H-diff* and *H-noise*. Loci with suggestive associations (5.0 × 10^−8^>*p*< 1.0 × 10^−5^) are shown in Supplementary Figure S1 and Supplementary Tables S8–S15.

#### 3.2 2 PAR regions

Among ∼45,000 genotyped and imputed variants in PAR regions, none reached genome-wide or suggestive significance in the combined or sex-specific analyses for the four ARHL traits (Supplementary Figure S3).

### 3.3 Heritability

The overall proportion of H-both variations explained by genotyped variants on the X chromosome was 0.4% [standard error (SE) = 0.8%, *p* = 3.5 × 10^−6^). The heritability estimates for the other ARHL traits were small and not statistically significant (*p* > 0.05).

### 3.4 *In-silico* mRNA expression analysis

We investigated mRNA inner ear expression for *MAP7D2/Map7d2* and *ZNF185/Zfp185* since they are new candidate genes for HL. Single-cell RNA (scRNA) experiments of the developing mouse cochlear epithelium show that *Map7d2* and *Zfp185* are both expressed in the cochlear epithelium in newborn mice (P1 developmental stage; Supplementary Figure S5). In addition, analysis of a scRNA dataset of mouse otic neuronal lineage cells (Sun et al., 2022) shows that *Map7d2* is also expressed in cochleovestibular ganglion cells at E13.5 (Supplementary Figure S5). *Zfp185* was not found to be expressed in otic neuronal lineage cells. Furthermore, in immortalized multipotent otic progenitor (iMOP) cells, *Map7d2* expression showed a 2.5-fold upregulation when cells were cultured in epidermal growth factor (EGF) (Supplementary Table S16). Finally, *MAP7D2* and *ZNF185* were also found to be expressed in the adult human inner ear (Supplementary Figure S6). For *LOC101928437,* a non-coding RNA, there are no human or mouse inner ear data available to evaluate its expression (no known orthologue).

## 4 Discussion

Analyzing UK Biobank data, we studied associations between X chromosome variants and ARHL. We identified three genes (*MAP7D2*, *LOC101928437,* and *ZNF185*) on the X chromosome associated with ARHL.

The genomic control lambda values for all analyses show slight genomic inflation (*λ* = 1.02–1.09, Supplementary Figures S2, S4), consistent with other genetic analyses of datasets with large sample sizes (Yang et al., 2011). Additionally, we are only reporting results for a single chromosome, and the X chromosome has more LD than the autosomes since recombination events only occurs in women outside of the PAR (Schaffner, 2004). There may also be polygenic effects which can lead to an increased *λ*_GC_ in the absence of genuine inflation (Yang et al., 2011).

We found an association between ARHL and *ZNF185*, which encodes an actin-cytoskeleton-associated Lin-l 1, Isl-1 and Mec-3 (LIM) domain-containing protein (Heiss et al., 1997). In keratinocytes and epidermis ZNF185 has been described as highly expressed in differentiating conditions, physically interacting with E-cadherin, a component of the adherens junctions, one of the critical cell-cell adhesive complexes crucial in the pluristratified epithelia (Smirnov et al., 2019). Although its role in the inner ear is unknown, we demonstrate it is expressed in the mouse cochlear epithelium and in adult human inner ear tissues (Supplementary Figures S5, S6). Furthermore, *znf185* was found as a scRNA-seq cluster marker (gene that distinguish the different cell clusters) of neuromast support cells in zebrafish *fgf3* mutants, which have increased hair cell regeneration (Lush et al., 2019).

In addition, we identified association between *MAP7D2* and *H-both* in the combined sex analysis. The combined OR for variants in *MAP7D2* was 1.03 (CI = 1.016–1.045). The MAP7 Domain Containing 2 (*MAP7D2*) is one of the members of the MAP7 family. It plays a vital role in regulating kinesin-1, promoting microtubule-based transport of numerous cellular cargoes entry into the axon (Hooikaas et al., 2019; Kikuchi et al., 2022). We demonstrate it is expressed in the cochlear sensory epithelium in mice, particularly in the inner and outer hair cells, in developing cochleovestibular ganglion cells and adult human inner ear tissues. In the latter, it is preferentially expressed in the cochlear duct which houses the sensory epithelium (Supplementary Figures S6). Furthermore, *Map7d2* expression is upregulated when otic progenitor (iMOP) cells are cultured in EGF, a growth factor important in the growth and differentiation of hair cells (Doetzlhofer et al., 2004). This suggests *Map7d2* may have a potential role in hair cell growth and differentiation.

Two variants in non-coding RNA, *LOC101928437*, were associated with *H-aid* in males. We could not evaluate inner ear expression of this non-coding RNA, since the data is unavailable. This non-coding RNA has been previously suggested to play a role in non-syndromic intellectual disability and shows a high level of brain specific expression (Zhou et al., 2015).

Although ARHL is a common disorder, little is known about the genetic susceptibility of this disorder. There is strong evidence of a difference in the prevalence of ARHL between males and females which is also observed in the UK Biobank (Wiley et al., 2008; Bowl and Dawson, 2019). Our results indicate that X chromosome SNVs are unlikely to be a factor in the observed higher prevalence in males than females. It has been previously suggested that these differences, at least partially, are due to hormonal pathways (Tadros et al., 2005; Hultcrantz et al., 2006; Souza et al., 2017).

One primary characteristic of the X chromosome in mammals is that female embryos undergo silencing of one of the two X chromosomes to equalize the dosage of X-linked genes between females and males (Lyon, 1961). This is extremely relevant to phenotypic changes, as mutations that could lead to severe complications in males are compensated by another chromosome in 50% of the female cells, which can confer protection against diseases (Cantone and Fisher, 2017). While many X-linked genes undergo X chromosome inactivation (XCI), some degree of expression heterogeneity among females has been reported: 15% of X-linked genes escape inactivation and 10% of X-linked genes exhibit variable patterns of inactivation (Carrel and Willard, 2005). Skewed XCI is present when one of the cell types is markedly in excess and may be a primary event due to chance or genetic factors (Puck and Willard, 1998) or secondary due to selection against or in favor of cells with a given genotype (Migeon and Haisley-Royster, 1998). Skewed XCI can cause phenotypic variability in both ways; it can either lead to a more severe phenotype or the expression of disease in females (Shoukat et al., 2020) or can act protective and reduce/avoid disease manifestations (Migeon, 2020). Skewed XCI is dependent on tissue and age (Zito et al., 2019), for example, the frequency of skewed XCI in peripheral blood cells increases with age, possibly due to selection (Hatakeyama et al., 2004; Kristiansen et al., 2005). Age-related X reactivation and escape XCI may also occur, and these processes could be related to neurological and oncological disease (Leppig and Disteche, 2001). It is known that skewing and escaping of XCI can occur in normal females and increases in tissues with age, therefore this process may also be involved in age-related diseases in females.

Large-scale volunteer databanks typically have limitations that include self-selection basis, no or limited numbers of children and older individuals, and study subjects mainly of European ancestry. We analyzed data from the UK Biobank for which the percent of females and average age is higher than that of the general UK population (Fry et al., 2017). HL phenotypes used in this study were self-reported. Unlike audiometric measurements, they can be less accurate and neither captured the severity nor the frequencies affected. Additionally, due to relatively small numbers of individuals of non-European ancestry, analysis was limited to white Europeans; therefore the results of this study may not be applicable to individuals from other ancestry groups.

In conclusion, we have identified significant associations between variants on chromosome X near *ZNF185, MAP7D2*, and *LOC101928437* with ARHL in white-Europeans. The identified variants need to be replicated in an independent sample. Additionally, it would be beneficial to determine if these genes play a role in ARHL in individuals of non-European ancestry. Although common variants on the X chromosome are involved in the etiology of ARHL, based on heritability estimates their role is likely limited.

## Data Availability

The original contributions presented in the study are included in the article/Supplementary Material, further inquiries can be directed to the corresponding author.

## Abbreviations

AR, age-related; HL, hearing loss; GWAS, Genome-wide association studies; SNP, single nucleotide polymorphism; PARs, pseudoautosomal regions; non-PAR, non-pseudoautosomal region; MAF, minor allele frequency; HWE, Hardy Weinberg Equilibrium; LD, linkage disequilibrium; InDels, insertion/deletions; H-aid, hearing aid; H-diff, hearing difficulty; H-noise, hearing noise; H-both, hearing difficulty and hearing noise; OR, odds ratio; CI, confidence interval; SE, standard error; XCI, X chromosome inactivation.
