# Supplementary material for: The genetic contribution of the X chromosome in age-related hearing loss
Source: Front Genet. 2023 Feb 21;14:1106328. doi: 10.3389/fgene.2023.1106328 (PMC9988903; doi:10.3389/fgene.2023.1106328)
Supplement: Supplementary file 2 [file Table1.DOCX]

**Supplementary Figures**

**The genetic contribution of the X chromosome in age-related hearing loss**

**Elnaz Naderi^1^, Diana M. Cornejo-Sanchez^1^, Guangyou Li^1^, Isabelle Schrauwen^1^, Gao T. Wang^1^, Andrew T. DeWan^2^, Suzanne M. Leal^1, 3*^**

^1^Center for Statistical Genetics, Gertrude H. Sergievsky Center, and the Department of Neurology, Columbia University Medical Center, New York, NY, USA.

^2^Department of Chronic Disease Epidemiology and Center for Perinatal, Pediatric and Environmental Epidemiology, Yale School of Public Health, New Haven, CT, USA.

^3^Taub Institute for Alzheimer’s Disease and the Aging Brain, Columbia University Medical Center, New York, NY, USA.

***Corresponding author:**

Suzanne M. Leal

sml3@cumc.columbia.edu

**
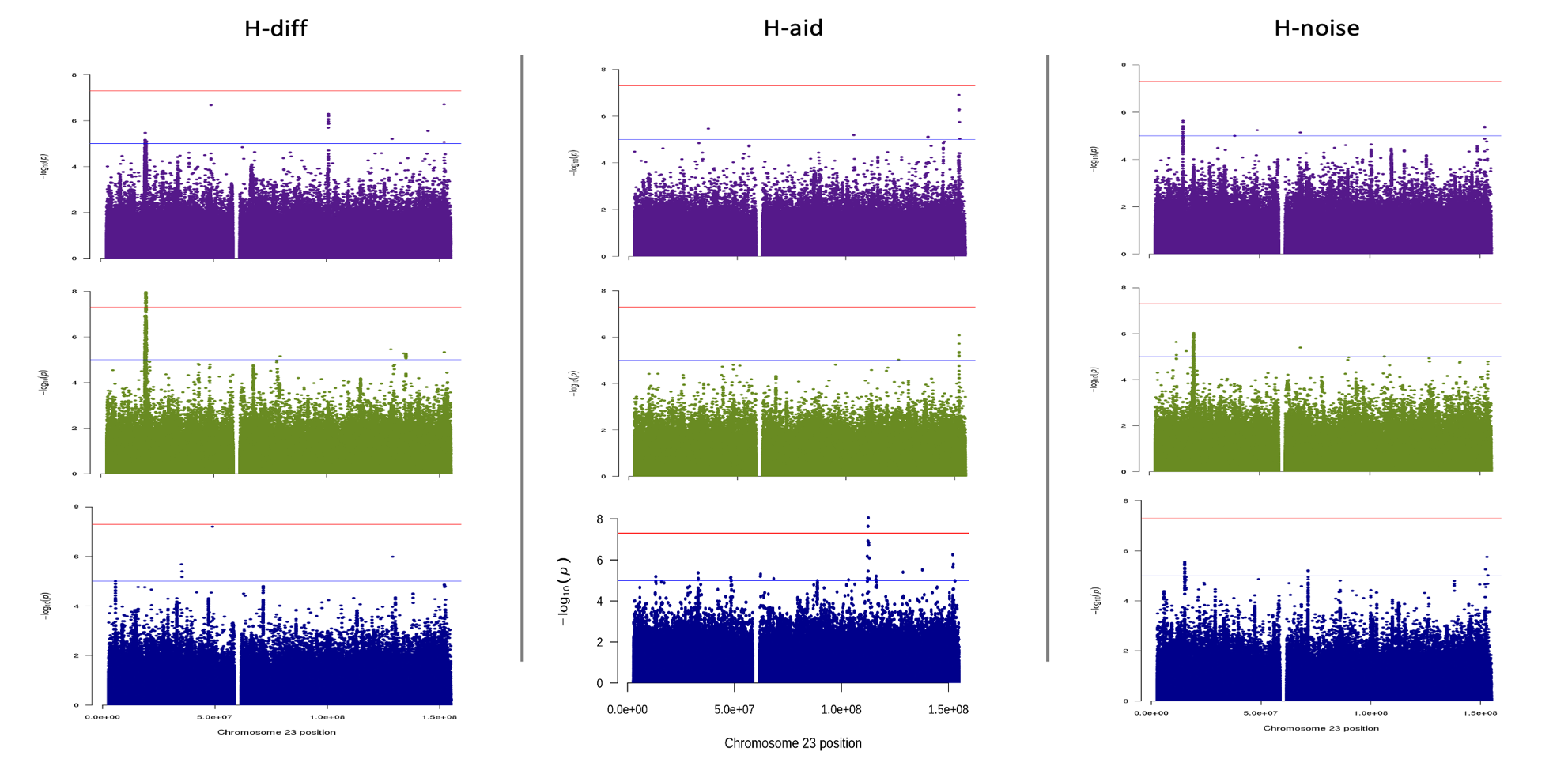
**

**Figure S1:** Manhattan plots for *H-noise, H-aid, and H-diff* (non-pseudoautosomal region)*.* Analyses: combined-sex (purple), females-stratified (green); and males-stratified (blue). The y axis displays −log_10_ p-values for each variant and the x-axis their position. The horizontal red and blue lines show the threshold for genome-wide significant (p=5×10^–8^) and suggestive (p=1×10^–5^) associations, respectively. The lambdas (λ_GC_) ranged from 1.025 to 1.069.


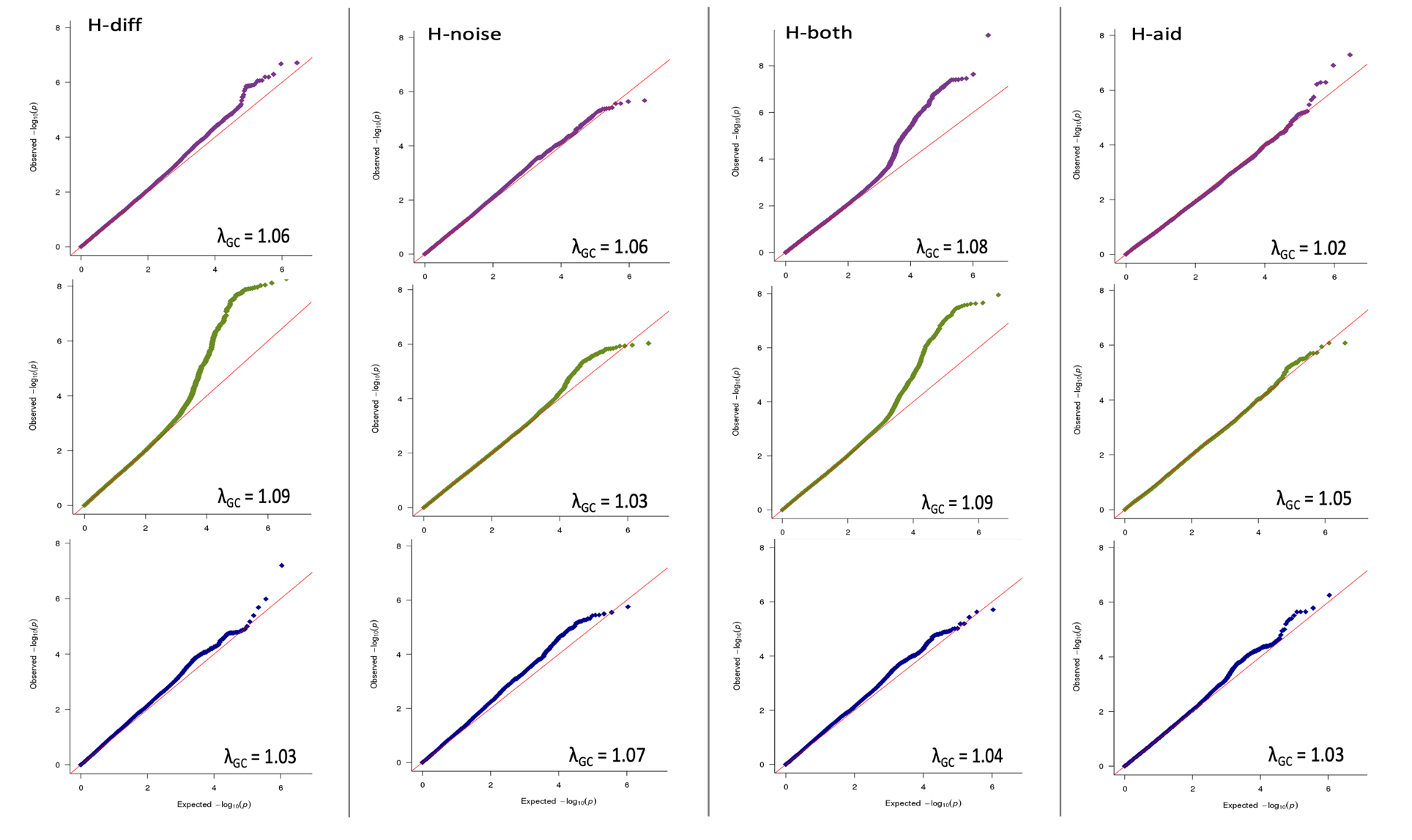


**Figure S2:** Q-Q plots for *H-aid, H-noise, H-both and H-diff* (non-pseudoautosomal region)*.* Analyses: combined-sex (purple), females-stratified (green); and males-stratified (blue). The y axis displays observed and the x-axis displays expected −log_10_ p-values for each variant, respectively. The lambdas (λ_GC_) ranged from 1.02 to 1.09.


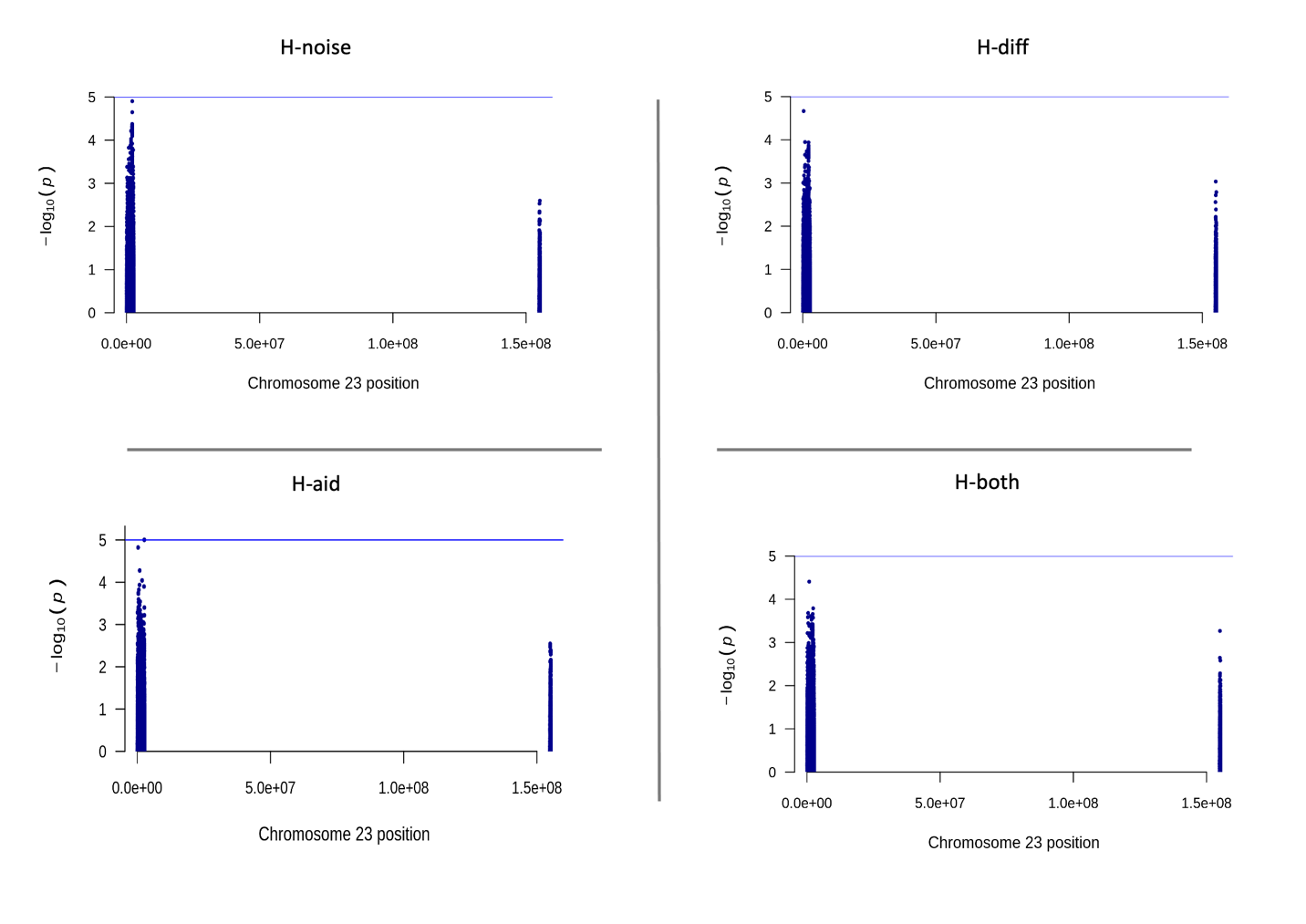


**Figure S3:** Manhattan plots for combined-analysis (females and males) for ARHL (pseudoautosomal regions)*.* The *y*-axis displays −log_10_ of the p-values for each variant site and the x-axis their position. The horizontal blue line displays the threshold for genome-wide suggestive (p=1×10^–5^) association. The lambdas (λ_GC_) ranged from 1.001 to 1.070.


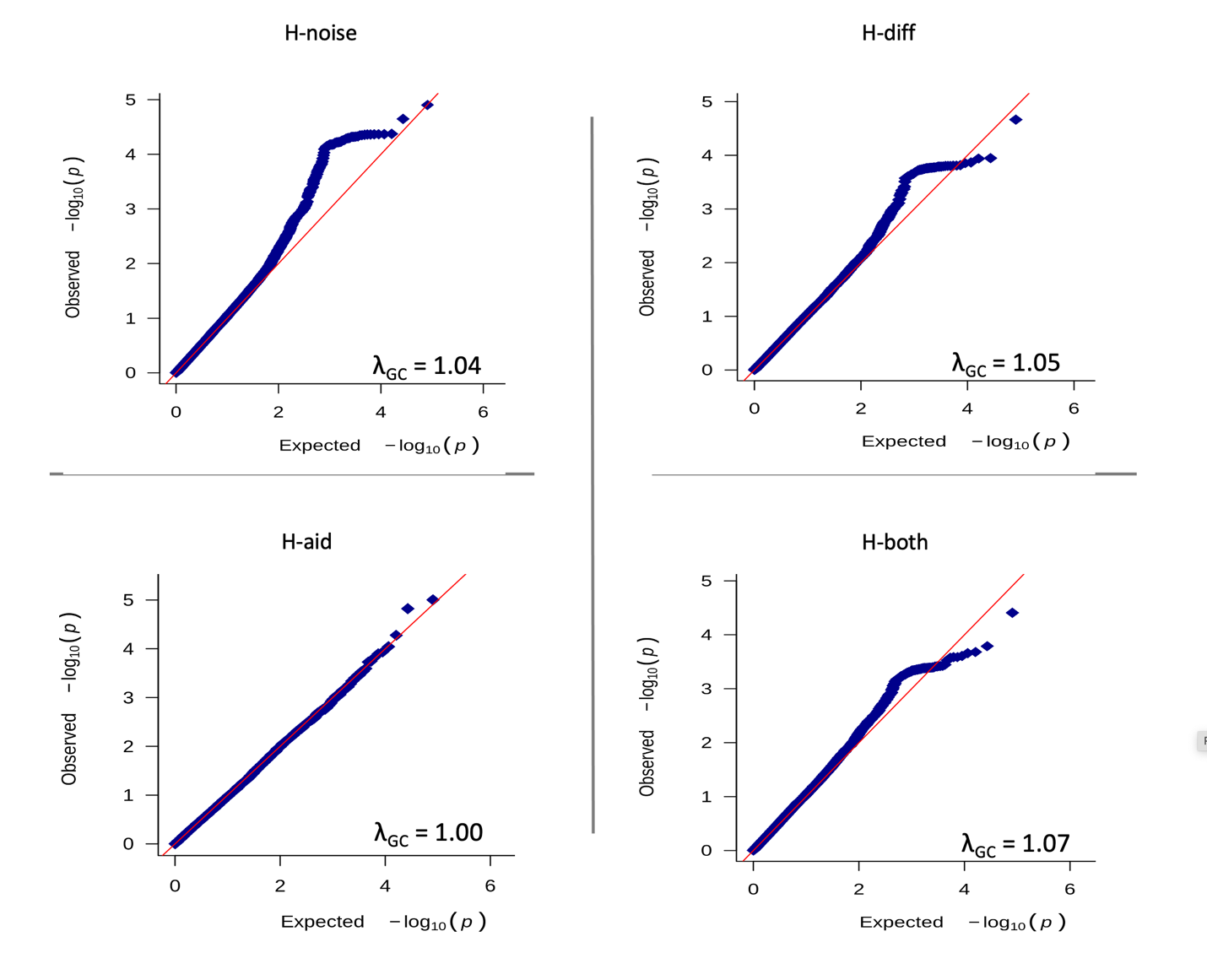


**Figure S4:** Q-Q plots for combined-analysis (females and males) for ARHL (pseudoautosomal regions)*.* The y axis displays observed and the x-axis displays expected −log_10_ p-values for each variant, respectively. The lambdas (λ_GC_) ranged from 1.00 to 1.07.

**
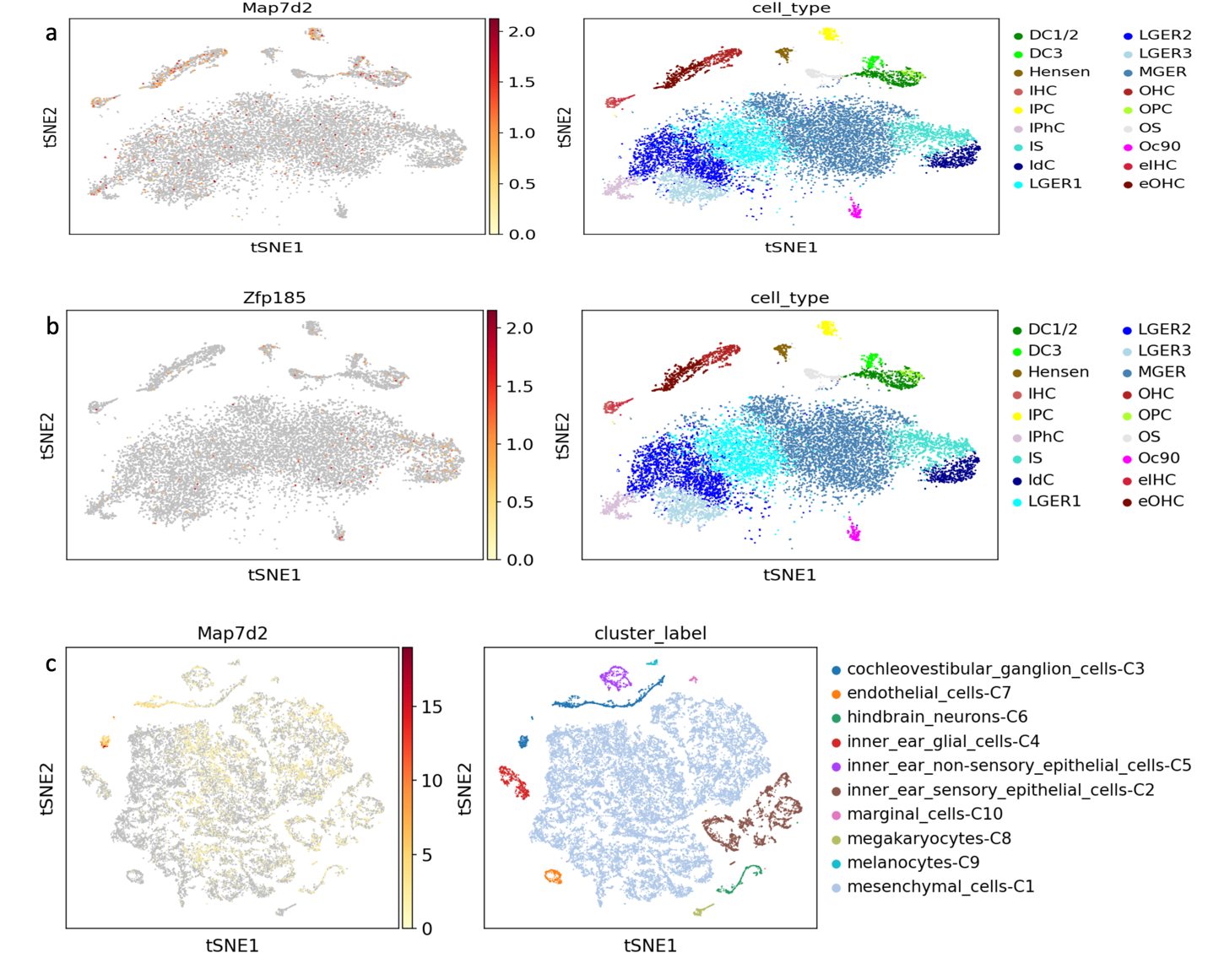
**

**Figure S5:** Expression of *Map7d2* and *Zfp185* in the mouse inner ear. Expression levels in the cochlear floor epithelium at P1 developmental stage for *Map7d2* **(a)** and *Zfp185* **(b)**, and expression levels for the cochleovestibular ganglion cells E13.5 stage for *Map7d2* ***(c)***. For the left panels higher number (red color) denotes high and lower number (yellow color) denotes low expression levels based on log transformed and normalized expression data. For the right panels the various colors indicate the cell type.

Abbreviations: DC1/2: Dieter Cells from rows 1 and 2; DC3: Dieter Cells from row 3; Hensen Cells; IHC: Inner Hair Cells; IPC: Inner Pillar Cells; IPhC: Inner Phalangeal Cells; IS: Inner Sulcus Cells; IdC: Interdental Cells; LGER1: Lateral Great Epithelial Ridge Cells, group 1; LGER2: Lateral Great Epithelial Ridge Cells, group 2; LGER3: Lateral Great Epithelial Ridge Cells, group 3; MGER: Medial Greater Epithelial Ridge Cells; OHC: Outer Hair Cells; OS: Outer Sulcus Cells; Oc90: Cells expressing Oc90; elHC: Less mature developing inner hair cells; eOHC: Less mature developing outer hair cells.


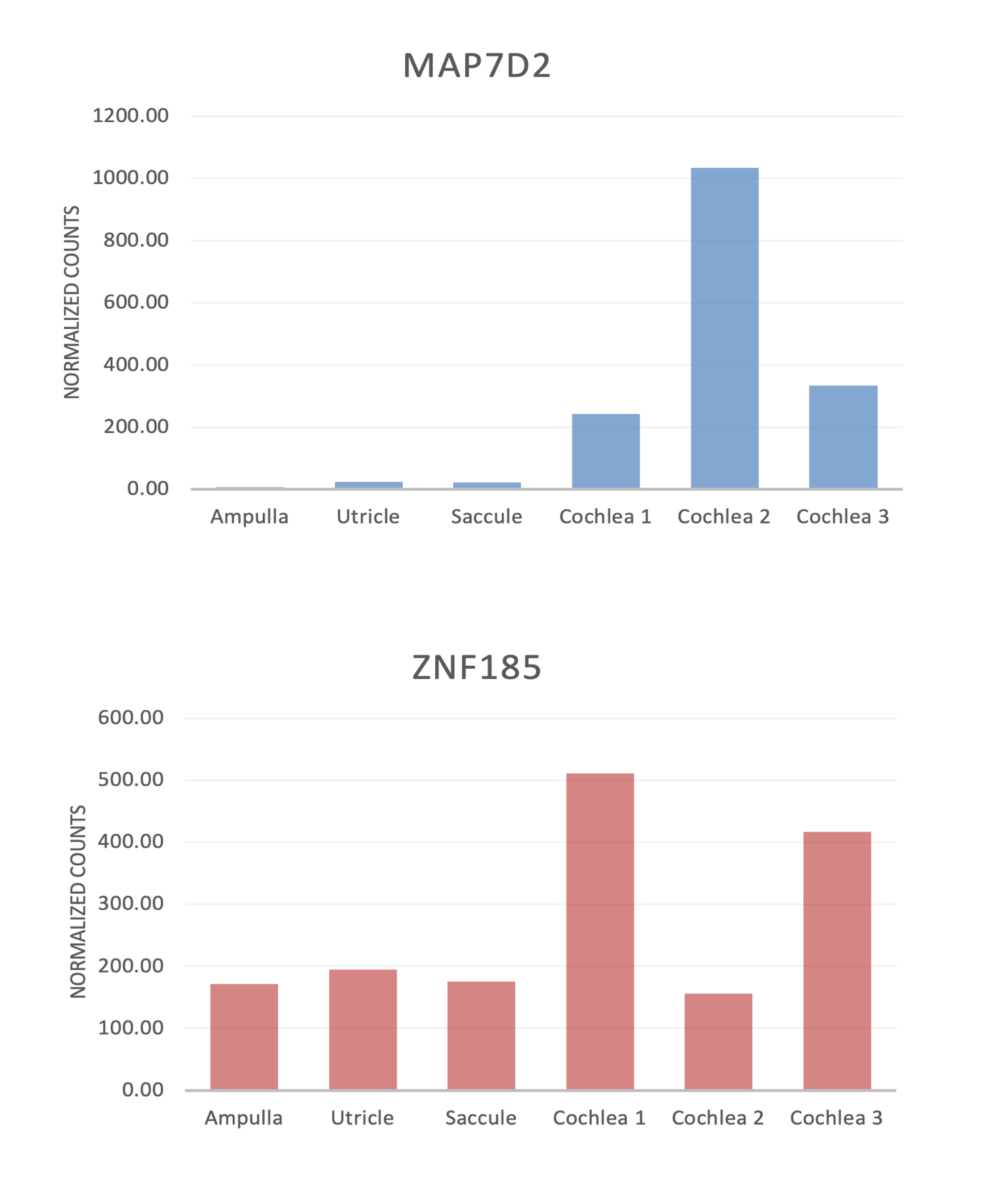


**Figure S6:** The adult human ear expression values of *MAP7D2* and *ZNF185* in normalized counts (y axis). mRNA expression in the cochlea was evaluated in cochlear duct tissue sampled from 3 individuals. In the vestibular system, RNA expression was assessed in 3 regions (Ampulla, Utricle, Saccule). ENSG00000184368 (MAP7D2) is preferentially expressed in the cochlea.
